# Including Functional Annotations and Extending the Collection of Structural Classifications of Protein Loops (ArchDB)

**Published:** 2009-11-24

**Authors:** Antoni Hermoso, Jordi Espadaler, E Enrique Querol, Francesc X. Aviles, Michael J.E. Sternberg, Baldomero Oliva, Narcis Fernandez-Fuentes

**Affiliations:** 1 Laboratori de Bioinformàtica, Institut de Biomedicina I Biotecnologia, Universitat Autònoma de Barcelona, Bellaterra 08193, Catalonia. Spain; 2 Laboratori de Bioinformàtica Estructural (GRIB), Universitat Pompeu Fabra/IMIM, Parc de Recerca Biomèdica de Barcelona, Barcelona 08003, Catalonia, Spain; 3 Structural Bioinformatics Group, Department of Biological Sciences, Imperial College, London SW7 2AZ, U.K; 4 Leeds Institute of Molecular Medicine, Section of Experimental Therapeutics, St. James University Hospital, Leeds LS7 9TF. U.K

**Keywords:** function annotation, loop structure classification, loop modeling

## Abstract

Loops represent an important part of protein structures. The study of loop is critical for two main reasons: First, loops are often involved in protein function, stability and folding. Second, despite improvements in experimental and computational structure prediction methods, modeling the conformation of loops remains problematic. Here, we present a structural classification of loops, ArchDB, a mine of information with application in both mentioned fields: loop structure prediction and function prediction. ArchDB (http://sbi.imim.es/archdb) is a database of classified protein loop motifs. The current database provides four different classification sets tailored for different purposes. ArchDB-40, a loop classification derived from SCOP40, well suited for modeling common loop motifs. Since features relevant to loop structure or function can be more easily determined on well-populated clusters, we have developed ArchDB-95, a loop classification derived from SCOP95. This new classification set shows a ~40% increase in the number of subclasses, and a large 7-fold increase in the number of putative structure/function-related subclasses. We also present ArchDB-EC, a classification of loop motifs from enzymes, and ArchDB-KI, a manually annotated classification of loop motifs from kinases. Information about ligand contacts and PDB sites has been included in all classification sets. Improvements in our classification scheme are described, as well as several new database features, such as the ability to query by conserved annotations, sequence similarity, or uploading 3D coordinates of a protein. The lengths of classified loops range between 0 and 36 residues long. ArchDB offers an exhaustive sampling of loop structures. Functional information about loops and links with related biological databases are also provided. All this information and the possibility to browse/query the database through a web-server outline an useful tool with application in the comparative study of loops, the analysis of loops involved in protein function and to obtain templates for loop modeling.

## Introduction

In a protein structure, loops are the regions of non-repetitive conformation connecting regular secondary structures, namely α-helices and β-strands. Loops are involved in protein function, stability and folding (Fetrow, 1995). They can play a wide repertoire of roles related to protein function: (i) recognition sites Complementary Determining Regions (CDRs) (Kim et al. 1999), (ii) protein-protein interactions: signaling cascades (Zomot and Kanner, 2003; Bernstein et al. 2004), dimerization (Fritz-Wolf et al. 1996), PDZ-motifs (Feng et al. 2003), (iii) ligand binding (p loop (Saraste et al. 1990) EF-hands (Kawasaki and Kretsinger, 1995), Nicotinamide adenine dinucletotide phosphate (NAD(P)) binding loops (Wierenga et al. 1986), glycin-rich-loop (Schenk and Snaar-Jagalska, 1999)), (iv) DNA-binding (helix-turn-helix motifs (Tainer et al. 1995), M13 phage (Coleman et al. 1986)); (v) forming enzyme active sites (e.g. Ser-Thr kinases(Johnson et al. 1998) or serine proteases (Wlodawer et al. 1989)). Moreover, loops play a vital role in correctly positioning catalytically important residues (Gunasekaran et al. 2003; Zgiby et al. 2002).

Experimental and theoretical evidences suggest that local structural determinants are frequently encoded in short segments of protein sequence. Local sequence-sequence-structure relationships derived from local structure/sequence analyses could significantly enhance the capacities of protein structure prediction methods (Yang and Wang, 2003). The reports of Shindyalov and Bourne (Shindyalov and Bourne, 2000), Lupas et al. (Lupas et al. 2001), and Tendulkar et al. (Tendulkar et al. 2004) suggest that folds are mainly made up of a number of simple local units of super-secondary structures, formed by few secondary structures connected by loops.

There is a large difference between known protein sequences (~2.4 millions; UniProt Release 5.0) (Bairoch et al. 2005) and protein structures (~30 000) (Berman et al. 2000). In the absence of an experimentally determined structure, *ab initio* and threading methods or comparative modeling methods can sometimes provide a useful 3D structure of a protein (Baker and Sali, 2001). Nevertheless, the recent improvements on the performance of fold prediction and homology modeling methods in successive CASP experiments (Venclovas et al. 2005) have not proved to be as successful as in loop model building. In general, these methods tend to correctly predict the protein core but not the loop regions. Errors in loops are the dominant problem in comparative modeling and often are the most difficult parts to model (Fiser et al. 2000; Burke et al. 2000). Thus, a database of structurally classified protein loops will have widespread applications (i.e. in model building or to complete locally undefined regions from an X-ray diffraction map).

The impact of loop modeling is significant. Currently, approximately 60% of all protein sequences can have at least one domain modeled on a related, known protein structure (Pieper et al. 2004). At least two thirds of the comparative modeling cases are based on less than 40% sequence identity between the target and the templates, and thus generally require loop modeling (Sanchez and Sali, 1998).

Structural genomics initiatives attempt to infer details of protein function via 3D structure determination (Eisenberg et al. 2000; Shapiro and Harris, 2000). If a new protein structure adopts a previously observed fold, functional details might be inferred by considering the function of other proteins adopting the same fold (Russell et al. 1997; Dietmann et al. 2002). If fold similarities are ambiguous or if a protein adopts a new fold, it is still possible to infer function by comparing key active site residues (Russell et al. 1998; Hegyi and Gerstein, 1999). Common structural motifs contain particularly useful information on the conservation of specific residues across species, being occasionally involved in the protein function (i.e. the activation loop of some kinases) or in the folding nucleus (Mirny and Shakhnovich, 2001).

Several works in loop classification have been published in the past years (Burke et al. 2000, Wintjens et al. 1996; Donate et al. 1996; Oliva et al. 1997; Wojcik et al. 1999; Oliva et al. 1998). However, these classifications were not web accessible or updated regularly. ArchDB (Espadaler et al. 2004; Oliva et al. 1997) has been updated since its creation, and the new version presented here includes three new classifications: ArchDB-95, ArchDB-EC and ArchDB-KI, plus the added value of functional annotations. The classification has been used to predict loop structures using the sequence profiles extracted from ArchDB (Oliva et al. 1998; Fernandez-Fuentes et al. 2005), studies of structure-function (Espadaler et al. 2006), and the extent of conservation of loop structures during evolution in protein kinases (Fernandez-Fuentes et al. 2004). The web-server provides an easy and efficient access to all the data. Users can query and retrieve the database in a number of ways (see below Browsing and Querying).

## Material and Methods

### PDB sets

**Figure 1.**
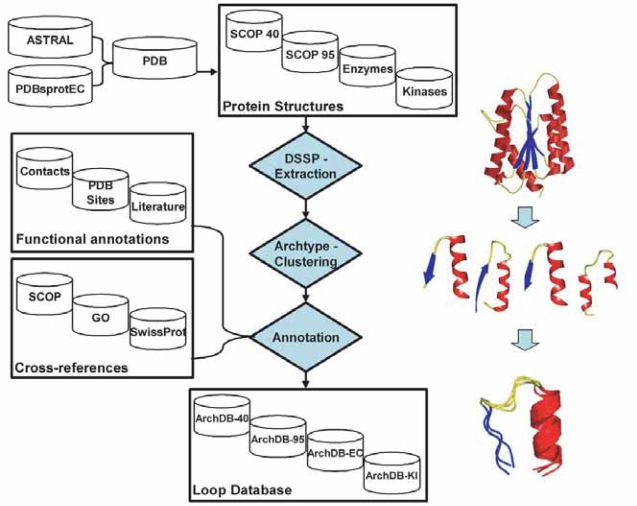
Overview of construction and annotation process of ArchDB. Four different PDB datasets were constructed to derive the four different classifications of loops. The process of the building of the database includes the extraction of loops, their clustering and annotation. A symbolical example is shown in the left side of the picture: starting form a protein structure, loops are extracted in form of structural motifs (secondary structure-loop-secondary structure), structurally clustered and finally annotated.

The current version of ArchDB contains 4 different types of loop structure classification of loops, namely:ArchDB-40,ArchDB-95,ArchDB-EC and ArchDB-KI each of them extracted from a different set of structures. ArchDB-40 is based on a list of protein domains of SCOP 1.67 (Lo Conte et al. 2002) with less that 40% sequence identity. ArchDB-95 is based in SCOP 1.67 (Lo Conte et al. 2002) using sequences with identity smaller than 95%. The two lists of protein domains were downloaded from ASTRAL compendium (Chandonia et al. 2002). ArchDB-EC is derived from a set of structures with known Enzyme Commission (EC) number (Kotyk, 1999) downloaded from http://www.bioinf.org.uk/pdbsprotec/(Martin, 2004). The program cd-hit (Li et al. 2002) was used to obtain a set of chain with less than 95% sequence identity. Finally, ArchDB-KI is derived from a set of structures with EC number 2.7.X.X (transferring phosphorus-containing groups) (Kotyk, 1999). See Figure 1 for a general overview of data collection and database building.

### Loop motifs extraction

The process of construction of the loop classifications is similar for the four sets included in ArchDB. First, structures not obtained by X-ray crystallography or with resolution larger than 3.0 Å are removed from the initial sets. The DSSP program(Kabsch and Sander, 1983) is used to locate loop segments, defining loops as fragments between any two regular secondary structures. The initial dataset of loops is further filtered by a quality rule: no loops were considered with missing residues or missing main chain atoms (including C_β_, except for Glycine).

### Clustering process

Loops extracted in the previous step are clustered according to structural similarity. The structural clustering of loops is obtained with an improved version (Espadaler et al. 2004) of the Arch-Type program (Oliva et al. 1997). In short, the clustering algorithm is based on a geometry comparison of the flanking secondary structures and on a density search on the [φ, ψ] space of the loop conformation. Geometry is defined by four internal co-ordinates of flanked secondary structures, a distance, D, between ending points and three angles: hoist, packing and meridian as shown in our previous work (Oliva et al. 1997). Two loop motifs share the same geometry if Δ (D, hoist, packing, meridian) belongs to the four-dimensional semi-open interval I = ((0, 0, 0, 0), (2, 45, 45, 45)] (Fernandez-Fuentes et al. 2006). The possible conformations of the loop fragment were defined by assigning the most accessible regions in [φ, ψ] space (Oliva et al. 1997). The regions are α, α_λ_, γ, β, β_p_ and ε (encoded by ArchType as “a”, “l”, “g”, “b”, “p” and “e”). Two special regions denoted “l/g” and “b/p” are defined as transition regions between the l and g conformations and between the b and p conformations, respectively. For a pair of loops, a conformational similarity score is obtained as the percentage of the total number of residues that can be equivalent with identical conformational codes.

Owing to the ±1 residue extension in loop length definition allowed because of the difficulty in defining the termini of the secondary structures and to the wide definition around [φ, ψ] regions in “l/g” and in “b/p” conformations, loops can cluster into more than one group. A re-clustering protocol has been devised to deal with the overlap between clusters. Overlapping clusters are merged depending on the percentage of shared loops. A cluster-membership p-value is calculated for each loop motif (see below Statistic significance of clusters). Overlapping clusters are merged if they have more than 80% of loops or if there is a common loop with membership p-value < 0.002 to both clusters. Averaged coordinates are recalculated and the process is repeated until convergence of the classification. The result is an optimized partition of the conformational space of loops that joints clusters (as obtained in Arch-Type(Oliva et al. 1997)) that contain structurally similar loops and a minimum overlap between subclasses.

## Results

### Database organization

ArchDB is structured into four levels of hierarchy: (i) at the *classification level*, there are links to the four loop classifications included in ArchDB: ArchDB-40, ArchDB-95, ArchDB-EC and ArchDB-KI; (ii) at the second level of the classification, loops were identified according to the bracing secondary structure type: α–α loops α–β loops, β α loops and β–β loops that are further split into β βhairpins (which are those loops between two β strands with at least one hydrogen bond between both strands) and β–βlinks, the complementary set in β–β loops; (iii) at *class* level, loops are grouped according to the loop length and [φ–ψ] loop conformation; and (iv) at *subclass* level the classes are subdivided according to the orientation of secondary structures or motif geometry. Each subclass is identified in ArchDB by a three-number code as defined in the original paper(Oliva et al. 1997). For instance, a subclass with a classification code αβ4.1.1 means that: it belongs to type α–β, it is the most populated class αβ4.1 with loops of length 4 ± 1 and it is the most populated subclass αβ4.1.1.

### Functional annotations

Subclasses have been classified as *putative structure/function-related subclasses* (PSFRS) or *functional subclasses* according to the degree of conservation of the annotations (DCA). The considered annotations have been obtained from: (i) SCOP identifiers; (ii) GO terms; (iii) EC codes among the original PDB chains; (iv) ligand contacts, i.e. residues found within a cut-off distance of 6Å from an hetero-atom, ligand, inhibitor, cofactor or complex partner molecule (protein or DNA) with the exception of D_2_O or crystallization buffer molecules; (v) PDB site information (residues identified by functional information from ACTSITE and SITE records in the PDB file header); and (vi) residues identified by the functional annotation collected from the literature and assigned to specific motifs (only for ArchDB-KI).

The functional annotation process is as follow. Each loop is annotated by its SCOP, EC and GO number. The conservation of these annotations is explored among the loops included in the same subclass. Three groups of DCA were defined: <50% conservation, between 50 and 75% conservation and >75% conservation. We define a subclass with more than 75% conservation of a given annotation as PSFRS. In case of ArchDB-KI, subclasses are considered functional subclasses when there is a meaningful conservation of functional residues in the loops of the cluster and more than 50% of its loops belong to proteins of the same SCOP superfamily. Besides the quantitative conservation of the SCOP, EC and GO numbers, a qualitative measure of potential function is also given if any loops included in the subclass have any annotation extracted from the PDB header (annotated as ACTSITE and SITE) and/or contacts with ligands. These features have been recently used on a method for protein annotation based on loop motifs (Espadaler et al. 2006).

### Current database content

The latest release of ArchDB contains a classification of 80,795 loop motifs, grouped into 4,758 *classes* and 8,462 subclasses (see Table 1 for complete details). The number of subclasses has increased by 40% when compared to previous release of ArchDB-40 (Espadaler et al. 2004). The most populated classification is ArchDB-95 that contains 36,153 loops in 2143 *classes* and 4,063 subclasses, covering ~47% of all loops found in SCOP, and includes loops up to 36 residues long, while ArchDB-40 contains 21647 loops in 1139 *classes* and 2550 subclasses. Regarding enzyme loops classifications: ArchDB-EC contains 20260 loops in 1338 *classes* and 2686 subclasses and ArchDB-KI has detailed functional information that has been manually curated; up to 76 out of the 203 subclasses (37%) contain residues with functional annotation collected from the literature.

### Browsing and querying

Users can browse through ArchDB data-sets or perform queries searching for loops motifs satisfying particular features:

- Belonging to a PDB structure by specifying the PDB identifier (Berman et al. 2000) or SWISS-PROT accession code (Bairoch and Apweiler, 2000);
- Browsing through ArchDB levels: i.e. classes and subclasses;
- Loop with particular bracing secondary structures type and geometry, loop size or loop [φ, ψ] conformation;
- Loops with a specific SCOP family, super-family and fold, SWISS-PROT keywords (Bairoch and Apweiler, 2000) or GO accession codes (Ashburner et al. 2000);
- Loops from subclasses with residues in contact with ligands and/or with PDB SITE annotations (and with bibliographical annotations for ArchDB-KI);
- PSFRS with DCA > 50%, between 50% to 75% or DCA > 75%;
- Sequence search. The search is performed on the selected classification using BLOSUM 62 (Henikoff and Henikoff, 1992) as mutation table to calculate the sequence score;
- Classes with the same conformation and subclasses with the same geometry and/or conformation of the loops of an uploaded protein structure. Structural classes and subclasses are assigned comparing the loop geometries and conformations of all the loops of an uploaded protein structure with the loops from the database. Secondary structure and loops of the uploaded coordinates of the query protein are defined with DSSP (Kabsch and Sander, 1983).

Points (iii) and (vii) will allow the user to obtain potential templates for loop modeling, as well as retrieving functional information about similar loops to check whether our loop could play a functional role or not. Analogously, for non-clustered motifs (single member subclasses), information described in points (iii), (v) and (vi) can also be retrieved. However, not all the structures classified in PDB databank (Berman et al. 2000) are represented in ArchDB. If a structure is not present in our classification, the PDB code(s) of the closest protein(s) in homology (i.e. the smallest e-value and the largest percentage of identity as aligned by PSI-BLAST (Altschul et al. 1997)) are shown.

Other type searches can be the list of motifs found in a given PDB structure, the list of subclasses satisfying specific features or the content of a given subclass. Structural and functional information for each PDB structure is accessible, including resolution, R-factor, PDB source, GO annotation, Enzyme annotation, and the SCOP domain classification.

**Figure 2.**
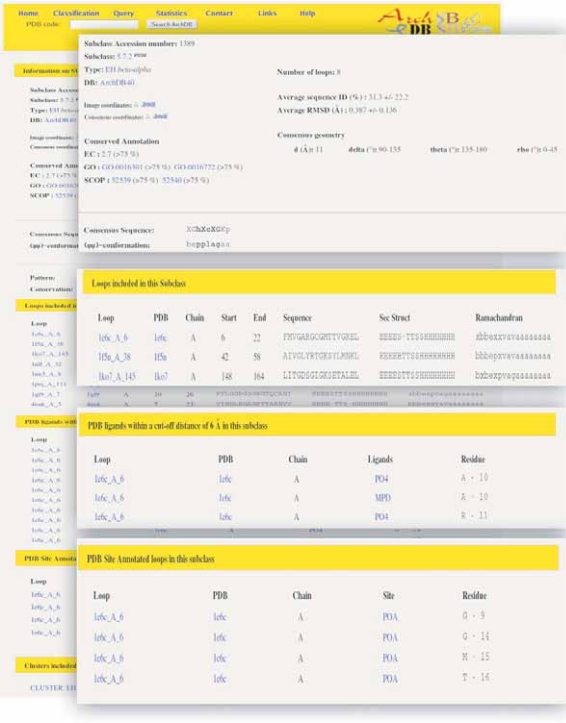
A snapshot of ArchDB website showing an example of a functional subclass: β–α 5.7.2, set ArchDB-40. Multiple alignments of sequence, secondary structure and conformation, position-specific residue conservation, ligand contacts within 6 Å, PDB site annotations are shown.

For each subclass, a table describing consensus features (sequence, geometry, percentage of sequence identity, averaged RMSD and its standard deviation) can be obtained. Additional information includes a PROSITE-like pattern (Falquet et al. 2002) with calculated position-specific entropy (Pei and Grishin, 2001) and a BLOSUM-like PSSM profile obtained with the multiple sequence alignment. 3D Images of superimposed motifs and averaged coordinates can be viewed using Rasmol (Sayle and Milner-White, 1995), Chime, Jmol or any molecular visualization program that can handle atomic coordinates in PDB format. Users can download coordinates for superimposed motifs or the average structure, which may be useful for loop reconstruction. Multiple alignments of sequences, secondary structures and [φ/ψ] conformations of the loops are provided. Information about residues in contact with ligands and residue with PDB site annotations (and with bibliographic annotations for ArchDB-KI) are also given, if any. See Figure 2 for a snapshot of a subclass page.

Finally, ArchDB is cross-linked to other important databases such as PDB (Berman et al. 2000), GO (Ashburner et al. 2000), SWISS-PROT (Bairoch and Apweiler, 2000) and SCOP (Lo Conte et al. 2002).

### Statistic significance of clusters

**Figure 3.**
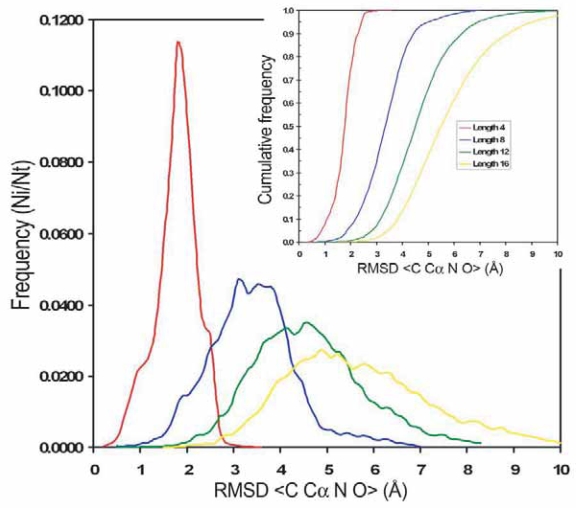
Observed frequencies of RMSD in the superposition between loops and random fragments extracted from PDB with lengths 4 (red), 8 (blue), 12 (green) and 16 (yellow). Inset: distribution functions of the frequencies of RMSD for the superposition of fragments. Calculated for fragments with lengths 4 (red), 8 (blue), 12 (green) and 16 (yellow).

RMSD is widely used as a measure to assess structural similarity between protein structures. However, the structural classification of loops into clusters is independent of the RMSD. We use RMSD to refine the subclasses by forcing the loops on the same subclass to share a similar conformation according to its RMSD. Small values of RMSD imply a meaningful similarity, but RMSD is highly dependent on the number of atoms being compared. To estimate the probability of observing a given RMSD, a random set of 50 loops motifs were selected for each loop length. Then, each loop was superimposed to 200 random PDB fragments of the same length selected from SCOP 40 (v.1.67). The density of probability of RMSD for fragment size 4, 8, 12 and 16 is shown in Figure 3. For all fragment sizes the distribution of values are Gaussian and centered around 1.9, 3.1, 4.7 and 5.2 Å, respectively.

**Figure 4.**
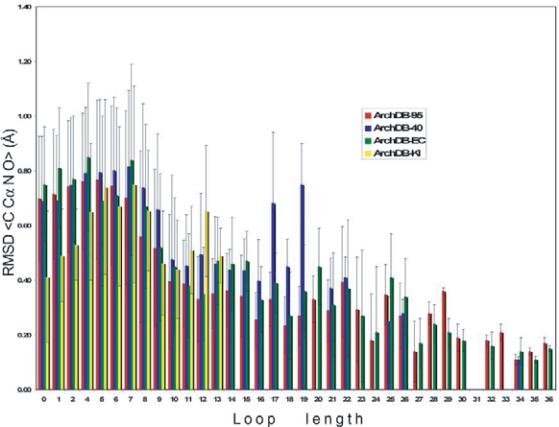
Subclasses averaged RMSD versus the loop length. The averaged RMSD of the sets of loop structures on each subclass was calculated with the main-chain atoms of the residues in the loop plus two bracing residues at each side. Additional extensions of the bars show the standard deviations of the averages. Shown for sets: ArchDB-95 (red), ArchDB-40 (blue), ArchDB-EC (green), and ArchDB-KI (yellow).

The function of distribution of RMSD for each loop size, defined as the probability to find a fragment with RMSD larger than a given value, allows us to calculate the expected p-value (Fig. 3 inset). The re-clustering algorithm yields a compact and accurate classification as it is shown in Figure 4. The average RMSD among loops that belong to the same subclass is small. For example, for length 8 and ArchDB40 classification, the average RMSD among loops that belong to the same subclass is 0.74 ± 0.31 Å (averaged RMSD ± standard deviation). The p-value of observing a RMSD of ~1 Å for fragments of size 8 is 0.0031.

## Discussion

The two major motivations for this study are: (i) to help to predict loop conformation in comparative modeling and, (ii) the availability of a functional annotated loop classification for the study of loops.

**Figure 5.**
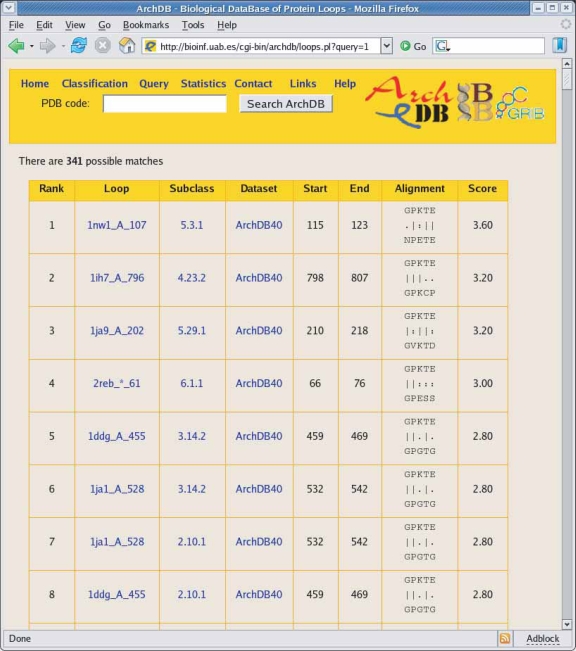
A snapshot of ArchDB website showing an example of a sequence search. A table sorted by sequence score and hyperlinks to ArchDB is given allowing users and easy and convenient examination.

We provide a classification of the conformation of loops with their associated sequence patterns and a PSSM profile for each structural alignment; together with the ability to search ArchDB database, provides a powerful tool to analyze loops in protein sequences. We have proved the usefulness of sequence profiles in loop structure prediction (Oliva et al. 1998; Fernandez-Fuentes et al. 2005). Figure 5 provides an example of template search for loop prediction (noted as a feature (vii) at the Browsing and Querying section). After entering the loop sequence and selecting the type of secondary structures that flank the loop and the classification on which to perform the search, the user receives a list of potential templates ranked by sequence score. The user can easily access the subclass pages using the hyperlinks provided and download the atomic coordinates of the template loop(s) and/or consensus coordinates if needed. Instead of searching for potential templates, users could be interested on functional annotated loops that are related with its query sequence by browsing among the functional annotations of the subclasses delivered with the sequence search (see below).

Functional annotated subclasses may help in the central problem of protein annotation. When sequence or structure comparisons fail to suggest a function, insights can come from discovery of functionally important local structural patterns. A subclass is a set of conserved local structural patterns. Conserved short stretches of amino acid sequences or motifs contain useful information on the conservation of specific residues involved in the protein function (catalysis or binding) or in the folding nucleus (Russell, 1998; Copley et al. 2001; Lupas et al. 2001; Mirny and Shakhnovich; 2001). The analysis performed on ArchDB-40 showed that up to 35% of active site residues are located in loops. An example of functional subclass is shown in Figure 2. In the subclass βα5.7.2 of ArchDB-40 more than 75% of the loops belong to the *P-loop containing nucleoside triphosphate hydrolases* SCOP superfamily. The EC number 2.7.-.- and the GO identifiers 0016301 and 0016772 are also conserved for more than 75% of the loops. Besides, some loops included in this subclass have contacts with ligands like phosphate, ADP and ATP analogs. Finally, four residues of one of the loops were annotated at the PDB header as ‘chain A walker A motif forming the p-loop which is the binding site for the phosphate of ATP’.

**Figure 6.**
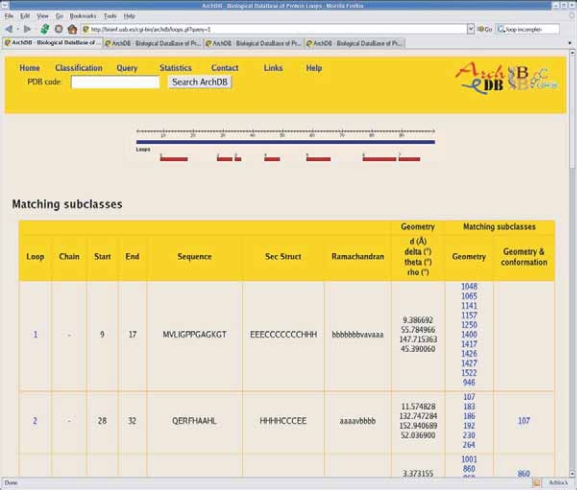
A snapshot of ArchDB website showing an example of a search using atomic coordinates. Loops are assigned using DSSP(Kabsch and Sander, 1983) and its location in the sequence is shown. Matching subclasses by loop geometry and matching subclasses by geometry and loop conformation are shown in a table jointly with hyperlinks to these subclasses.

We can use functional annotated subclasses to search for matches of loops in a newly determined structure and thereby suggest putative function or bindings. It can be of special interest given the pace of structures production on structural genomic initiatives worldwide, where functional insights can come from discovery of functionally important local structural patterns. For that reason, we created ArchDB*-EC*, a subset of ArchDB restricted to structures from proteins with known enzymatic function. ArchDB-EC is aimed at users focusing on loops involved in active sites. We expect this subset to be of interest when searching for loops with catalytic roles in protein structures. Figure 6 shows an example of a search using the loops extracted from a structure (noted as feature (viii) at the Browsing and Querying section). After uploading a protein structure, ArchDB extracts all loops and structurally compare with the classes and subclasses (and single loops if selected) classified. All the hits are presented in a table with the hyperlinks to the subclasses pages. Users can easily explore and browser the results and assess the significance of the results to their specific queries. In addition, this type of search yields all possible loop conformations that bridge two secondary structures. Users could be interested on comparing its own loop conformation with alternative ones (i.e. structural models, alternative loop conformations in catalytic/mobile loops, etc.).

**Figure 7.**
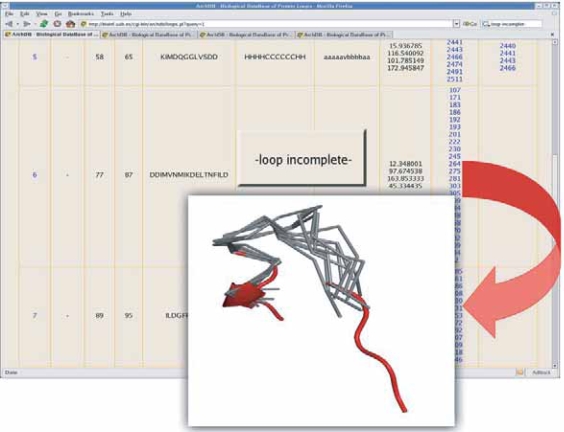
A snapshot of ArchDB website showing an example of search using a protein structure where one of the loops is incomplete. This loops is annotated as ‘*-loop incomplete-*’. The superposition between the query loop, depicted in cartoon representation and red color, and consensus structure of the candidate subclasses, represented in Cα trace and grey color, is shown in the inset figure. The structure representation of the atomic coordinates was produced using PyMOL (http://pymol.sourceforge.net/).

On the other hand, the search using protein structures can be also used for loop modeling. All subclasses that fit the geometry of the adjacent secondary structures of a motif can be retrieved from ArchDB. Consequently, for a missing or wrongly modeled loop region, users can download the atomic coordinates of the subclasses and superimpose them to the known framework (see Fig. 7). Broken or missing loops are shown as ‘*-loop incomplete-*’ at the result table if the loop region was missing while a list of compatible subclasses according to motifs-geometry is provided. This feature is also applicable in case of structural models, namely structures predicted by computational means. Users might be interested on searching for loops that can span a fixed core (i.e. secondary structure elements) obtained by comparative modeling, threading, or an *ab initio* prediction.

Other aspects of protein structure prediction could benefit from this classification. The preferred sequence motifs for loops could be used to improve the accuracy of secondary-structure prediction. The loop sequence motifs could be used to refine the boundaries of the predicted secondary structures. Furthermore, secondary-structure prediction can be used as the starting information for fold recognition (Fischer and Eisenberg, 1996; Hvidsten et al. 2003; Koretke et al.). The assembling of short fragments from known structures has been a widely used approach to construct protein structures. Recently, Kolodny et al. (Kolodny et al. 2002), Kolodny and Levitt (Kolodny and Levitt, 2003), Yang and Wang (Yang and Wang, 2002), Du et al. (Du et al. 2003) and Fernandez-Fuentes et al. (Fernandez-Fuentes et al. 2006) have employed short protein fragments to build protein structures.

## Conclusions

We described an up-to-date and exhaustive classification of loop structures. The database is composed of four different classifications customized for specific requirements and includes functional annotations. We built a flexible search engine that allow the querying/browsing of the database in a number of ways, either using sequence, structure, and feature-based information. All this classified data and the wide range of possibilities of the search engine shapes a powerful tool with applications in different areas of biological sciences and bioinformatics.

In our previous works we proved that loop classifications are suitable tools for loop structure prediction, in the specific case of Immunoglobulin loops (Oliva et al. 1998) or in loops in general (Fernandez-Fuentes et al. 2005). Also we have verified the conservation of loop structures related with its function (Espadaler et al. 2006) and the extent of conservation of loop structures during evolution in the specific cases of protein-kinases (Fernandez-Fuentes et al. 2004). In summary, we provided a high quality and functional annotated loop database with proved usefulness in protein structure and function prediction.

## Availability and Requirements

A web-server to browse and query ArchDB is available at http://sbi.imim.es/archdb. All the data is stored in *MySQL* tables and we use DBI-DBD (DataBase Interface-DataBase Driver) and related modules for communication between the scripts and the *MySQL* database server. We use a CGI (Common Gateway Interface) module to create the HTML (HyperText Markup Language) output.

No specific requirements are needed to browse/query ArchDB, however, users need molecular visualization programs such as Pymol (http://pymol.sourceforge.net/) or Rasmol (Sayle and Milner-White, 1995), or web-browser pluggings such as Chime (http://www.mdl.com/products/framework/chime/) or Jmol (http://jmol.sourceforge.net/), to visualize loop structures. The database and web-server are freely accessible without any restriction for academic use.

## Figures and Tables

**Table 1.** Total of classes, subclasses and loops classified. Table showing the total of loops, classes, and subclasses of the proteins from ArchDB-95, ArchDB-40, ArchDB-EC and ArchDB-KI. Subclasses annotated as functional subclasses in ArchDB-KI are shown between parentheses. Also, *putative structure/function-related subclasses* (PSFRS) are indicated in parentheses for ArchDB-95, ArchDB-40 and ArchDB-EC.

		**Loop type**				
		α**–**α	α**–**β	β**–**α	ββ**-links**	ββ**-hairpins**
ArchDB-40 (3640 pdbs)	Loops	3856	3528	5218	2771	6274
	Classes	192	185	304	229	209
	Subclasses	526 (233)	460 (249)	733 (520)	433 (228)	398 (206)
ArchDB-95 (5472 pdbs)	Loops	6171	7390	7835	5468	9289
	Classes	398	370	532	437	405
	Subclasses	840 (349)	843 (512)	1090 (623)	707 (321)	543 (232)
ArchDB-EC (2349 pdbs)	Loops	3075	6340	4701	2017	4127
	Classes	241	320	381	221	175
	Subclasses	488 (191)	720 (349)	773 (275)	367 (107)	338 (112)
ArchDB-KI (134 pdbs)	Loops	693	682	767	368	245
	Classes	40	36	30	21	12
	Subclasses	51 (19)	65 (15)	46 (30)	29 (8)	12 (4)

## Abbreviations:

Å: angstrom;
CASP: critical assessment of structure prediction;
DCA: degree of conservation of the annotations;
DSSP: dictionary of protein secondary structures;
EC: enzyme nomenclature;
GO: gene ontology;
PDB: protein data bank;
PSFRS: putative structure/function-related subclasses;
PSSM: position specific scoring matrix;
RMSD: root mean square deviation;
SCOP: structural classification of proteins.
